# Incidence, Causes and Outcomes of Postpartum Hemorrhage in Eastern Ethiopia: A Multicenter Surveillance Study

**DOI:** 10.1007/s10995-024-03986-4

**Published:** 2024-09-19

**Authors:** Sagni Girma, Abera Kenay Tura, Redwan Ahmed, Marian Knight, Thomas van den Akker

**Affiliations:** 1 College of Health and Medical Sciences, Haramaya University, Harar, Ethiopia; 2 Department of Obstetrics and Gynaecology, Leiden University Medical Centre, Leiden, The Netherlands; 3 Department of International Public Health, Liverpool School of Tropical Medicine, Liverpool, UK; 4 Department of Obstetrics and Gynecology, Hiwot Fana Specialized University Hospital, Harar, Ethiopia; 5 National Perinatal Epidemiology Unit, Nuffield Department of Population Health, University of Oxford, Oxford, UK; 6 Athena Institute, Vrije Universiteit Amsterdam, Amsterdam, The Netherlands

**Keywords:** Postpartum hemorrhage, Obstetric surveillance, Maternal death, Ethiopia

## Abstract

**Objectives:**

Maternal mortality remains an unfinished global agenda and postpartum hemorrhage (PPH) remains one of the leading causes. The aims of this study were to describe the incidence, underlying causes, and case fatality rate of PPH in public hospitals in eastern Ethiopia.

**Methods:**

This study was part of a larger Ethiopian Obstetric Surveillance System (EthOSS) project — a multicenter surveillance of women admitted to 13 public hospitals in eastern Ethiopia due to any of the five major obstetric conditions: obstetric hemorrhage, eclampsia, uterine rupture, sepsis, and severe anemia – conducted from April 1, 2021 to March 31, 2022. All registers in maternity units of those hospitals were reviewed to identify eligible women and collect data on sociodemographic and obstetric characteristics, management and maternal outcomes at discharge or death. Findings were reported using descriptive statistics.

**Results:**

Among 38,782 births registered during the study period, 2043 women were admitted with at least one of the five major obstetric conditions. Of these 2043, 306 women (15%) had PPH corresponding with an incidence rate of 8 (95% CI: 7–9) per 1000 births. Uterine atony was the main underlying cause in 77%; 81% of women with PPH received at least one uterotonic drug, and 72% of women for whom blood was requested received at least one unit. Of the 70 hospital based maternal deaths, 19 (27%) died from PPH, making a case fatality rate of 6 per 100.

**Conclusions:**

Although the overall incidence of PPH appeared low, it was still the underlying cause of death in one out of four women who died. The contributing factors might be that one in five women with PPH did not receive any uterotonic drug and the low blood transfusion. Ongoing audit, followed by targeted action, is essential to improve care quality and reduce adverse maternal outcome. The relatively low incidence may reflect under-recording in paper-based records, implying that further research into methods to optimize the surveillance is needed.

**Supplementary Information:**

The online version contains supplementary material available at 10.1007/s10995-024-03986-4.

## Significance

What is Already Known on this Subject?

What this Study adds?

Although PPH remains the leading cause of maternal mortality in many low-resource settings, systems to track progress and effects of interventions to reduce its burden are lacking. Identifying the incidence, underlying contributing factors and management outcomes would assist in devising tailored interventions.

While an inadequate administration of uterotonics and low blood transfusion could have contributed to PPH causing significant proportion of maternal death in the study settings, audit of care, context specific interventions and improving paper based recording and research to optimize the surveillance need due attention.

**Supplementary Information:**

The online version contains supplementary material available at 10.1007/s10995-024-03986-4.

## Introduction

Although the global maternal mortality ratio (MMR) is declining, it remains disproportionately high in low-and-middle-income countries. In 2020, sub-Saharan Africa (SSA) was the region with the highest MMR globally, estimated to be 545 per 100,000 live births accounting for more than 70% of the global 287,000 maternal deaths (World Health Organization, 2023; Zarocostas, 2023). Likewise, although Ethiopia’s MMR of 267 per 100,000 live births showed a one-third reduction from its level in 2016 (401 per 100,000), an estimated 10,000 annual maternal deaths occurred in Ethiopia in 2020 (World Health Organization, 2023b). Moreover, for each maternal death, 20 or 30 women develop severe obstetric complications, but survive (Ashford, 2002; Firoz et al., 2013).

Postpartum hemorrhage (PPH), commonly defined as an excessive bleeding from the genital tract following the birth of the neonate (> 500 ml in case of vaginal birth or > 1000 ml in case of cesarean section), remained one of the leading causes of maternal deaths in Ethiopia (Federal Ministry of Heath of Ethiopia, 2021; World Health Organization, 2018). Although PPH affects about 5% of global births (Carroli et al., 2008; Mavrides, 2016; Souza et al., 2013; World Health Organization, 2012, 2018), it is the leading cause of maternal deaths in many low- and middle-income countries (Say et al., 2014). Preventing PPH related deaths and complications requires early recognition and management (Andrikopoulou & D’Alton, 2019; Higgins et al., 2019; Mavrides, 2016; World Health Organization, 2012). In Ethiopia, PPH has been reported to complicate 3 to 17% of all births, with considerable variation depending on context, facility type, study design and case definition (Achamyelesh & Nebiyu, 2021; Amanuel et al., 2021; Asrat & Shimeles, 2021; Bewket et al., 2022a, Bewket et al., 2022b; Biruk et al., 2019; Habitamu et al., 2019; Jemberu et al., 2022; Sinetibeb et al.,2021; Tadesse et al., 2022). In 2020, 47% of maternal deaths in Ethiopia were due to hemorrhage (Ethiopian Public Health Institute, 2020).

Whilst the contribution of PPH to maternal deaths and complications in Ethiopia is well-documented in the literature, a robust and efficient system for continuously monitoring the frequency of PPH is not currently in place (Amanuel et al., 2021; Bewket et al., 2022a; Jemberu et al., 2022; Sinetibeb et al., 2021; Temesgen, 2017). This hampers rapid evaluation of efforts to reduce the incidence of such conditions. Lack of robust numbers, including the number of women who have PPH but survive, hampers a comprehensive understanding of the burden of PPH and its case fatality rate (CFR).

It is noteworthy that many high-income countries now have obstetric surveillance systems for monitoring progress and effects of interventions. The UK Obstetric Surveillance System (UKOSS), for instance, has demonstrated importance in improving clinical care and maternal outcomes in the UK and beyond (Knight et al., 2005; Knight & Lindquist, 2013). Based on UKOSS methodology, the Ethiopian Obstetric Surveillance System (EthOSS) was established in April 2021 in hospitals with maternity units in eastern Ethiopia through prospective registration of five major obstetric conditions: obstetric hemorrhage, eclampsia, uterine rupture, sepsis, and severe anemia (Abera et al., 2023a). This study aimed to describe the incidence, underlying causes, and the CFR of PPH among women admitted to any of the 13 public hospitals in eastern Ethiopia with any of the five major conditions as part of the EthOSS project.

## Methods

### Study Settings

This study was conducted as part of the EthOSS project, a study covering a network of 13 public hospitals with maternity units from April 1, 2021 to March 31, 2022. EthOSS is a multicenter study established for monitoring pregnancy outcomes among women hospitalized during pregnancy, childbirth or within 42 days postpartum in eastern Ethiopia. In these 13 public hospitals, 38,782 women gave birth during the one-year study period. Details of the EthOSS methodology, type or level of hospitals included and characteristics of women admitted were described elsewhere (Abera et al., 2023a, b). In brief, data on five priority obstetric conditions—obstetric hemorrhage, eclampsia, uterine rupture, sepsis, and severe anemia—were registered daily by designated clinicians in the consenting hospitals followed by detailed data collection by EthOSS research assistants at the end of each reporting month. Based on the 2019 Ethiopian mini-demographic and health survey, only half (48%) gave birth in health facilities in Ethiopia, ranging from 41 to 69% in different areas in eastern Ethiopia. In this study, we focused on women admitted for PPH to any of these hospitals.

### Study Design, Population and Data Collection

This was a multicenter cohort study. All women who were admitted to any of the 13 participating hospitals during pregnancy, childbirth or within 42 days postpartum were the source population whereas the study population were women who were admitted for PPH (and who either gave birth at the same hospital or were referred from other facilities or came in after home birth) in any of these 13 hospitals. PPH was defined as excessive bleeding following childbirth (> 500 ml following vaginal birth or > 1000 ml following cesarean section) (Federal Ministry of Health of Ethiopia, 2021a; World Health Organization, 2018) recorded based on the diagnosis documented by the managing clinician in the woman’s case file. We reviewed records of all women admitted with PPH and all registers in the labor and gynecology wards, operating theatres and the intensive care units of each hospital.

At each hospital, a designated midwife recorded the total number of women admitted with PPH along with their medical registration numbers and date of admission or date of diagnosis daily. At the end of each month, the midwife reported the number of women with PPH and their details to the EthOSS team in a protected online system: the Kobo Toolbox. After receiving the reports, EthOSS research assistants visited respective hospitals to screen the medical records of each woman for eligibility (if the case file is truly of PPH, in this case) and collected detailed data on socio-demographic characteristics, past obstetric history, underlying causes, management (active management of third stage of labor, use of uterotonics, manual and surgical interventions) and outcomes of PPH. Causes of PPH and interventions given were picked as documented in the charts by the managing clinicians. These data were entered into Kobo Toolbox offline and later uploaded to the central EthOSS database of the Kobo Toolbox system, which is password-protected and only accessed by the EthOSS investigators. The data abstraction form used in EthOSS was adapted from the UKOSS data abstraction forms (Knight et al., 2005) and the Ethiopian Maternal and Perinatal Death Surveillance and Response (MPDSR) guideline (Ethiopian Public Health Institute, 2017).

### Data Processing and Analysis

The data were exported to and analyzed using Stata 14 (Statacorp: College Station, Texas 77845 USA). Missing values and outliers were checked by running frequencies, and cross tabulations. Descriptive statistics was used to present results in tables, figures, graphs, and charts as appropriate. Incidence of PPH was calculated as the number of women admitted for PPH per 1000 births, and CFR was defined as the proportion of women who died from PPH among all women hospitalized for PPH.

#### Ethics Approval

The study was conducted in accordance with the Declaration of Helsinki. The study protocol was reviewed and approved by the Institutional Health Research Ethics Review Committee (IHRERC) of the College of Health and Medical Sciences of Haramaya University, Ethiopia (Ref No. IHRERC/024/2021); and the University of Oxford’s Oxford Tropical Research Ethics Committee (OxTREC); (Reference 530 − 21). Informed consent for the study was obtained from medical director and head of maternity units of each hospital. This study is based on review of case files and therefore the need for consent from woman was waived.

#### Consent to Participate

This study is based on a review of case files after a woman’s discharge or death, and therefore the need for consent from the individual woman was waived. The facility’s consent to be part of the study was signed by the medical director and head of the maternity unit of each hospital. Data collectors also signed data privacy consent forms to keep confidentiality of the information they accessed during data abstraction.

## Results

### Socio-demographic Characteristics

In the one-year period, 38,782 births and 34,090 live births were registered in the 13 hospitals participated in the EthOSS. A total of 2043 women (including 70 women who died) were admitted to these hospitals with at least one of the five EthOSS conditions (2043/38,782; 5%), 306 (15%) had PPH. Among the 306 women with PPH, 234 (76%) gave birth at a health facility, 43 (14%) were < 20 years of age and another 43 (14%) were aged 35 years or above; 226 (74%) had no antenatal care (ANC) consultations attended. Slightly more than half were primiparous (158; 52%). We found that six of 306 women (2%) were admitted to an intensive care unit (ICU) for critical care (rather than for mere recovery after cesarean section) (Table 1). Of the six ICU admissions, three were in a tertiary/academic hospital, one in a referral hospital, one in a general hospital and one in a primary hospital. Tertiary and referral hospitals have a well-established ICU with mechanical ventilation, and well-trained anesthesiologists and emergency and critical care doctors, whereas the availability of ICU equipment and trained health workers are rare or none at all in general and primary hospitals. None of these three hospitals where ICU admissions happened had any renal replacement therapy available.

**Table 1:** Basic socio-demographic and obstetric characteristics of women admitted with PPH to hospitals in eastern Ethiopia, 2022 (n = 306)

Variables	Category	Frequency(n)	Proportion(%)
Age in years	< 20	43	14
	20–34	220	72
	> 35	43	14
Parity	Primiparous	34	11
	Multiparous	158	52
	Not specified	114	37
ANC consultations	Attended any ANC consultations	77	25
	No ANC consultations attended	226	74
	Not specified	3	1
Onset of labor	Spontaneous	214	70
	Induced	18	6
	CS before labor	8	3
	Not specified	66	21
Mode of birth	Vaginal	281	92
	CS	17	5
	Not specified	8	3
Status of neonate at birth	Alive	199	65
	Stillbirth	45	15
	Not specified	62	20
Place of childbirth	Health facility	234	76
	Home	67	22
	Not specified	5	2
Admission to ICU	Yes	6	2
	No	300	98

*Keys*: ICU, intensive care unit, ANC, antenatal care; CS, cesarean section; PPH, postpartum hemorrhage

Of the 306 women with PPH, 132 (43%) were admitted to eight general hospitals, followed by 89 (29%) to a tertiary/academic hospital, and 31(10%) to a referral hospital. Nine out of 19 PPH-related maternal deaths happened in one tertiary/academic hospital (Table 2).

**Table 2:** Basic demographic and clinical characteristics of women admitted with PPH by level of hospitals in eastern Ethiopia, 2022 (n = 306)

Characteristics	Level of hospitals				
	Tertiary/academic	Referral	General	Primary	Total
Total PPH cases admitted	89	31	132	54	306
Referred or came after birth	82	21	62	16	181
Gave birth in the same facility	77	24	97	36	234
Total alive neonates	64	25	75	35	199
Total still births	22	2	18	3	45
Vaginal birth	82	29	123	47	281
Caesarean section	7	1	6	3	17
Number of women who died	9	2	6	2	19

### Incidence, Causes, and Management of PPH

**Fig. 1:**
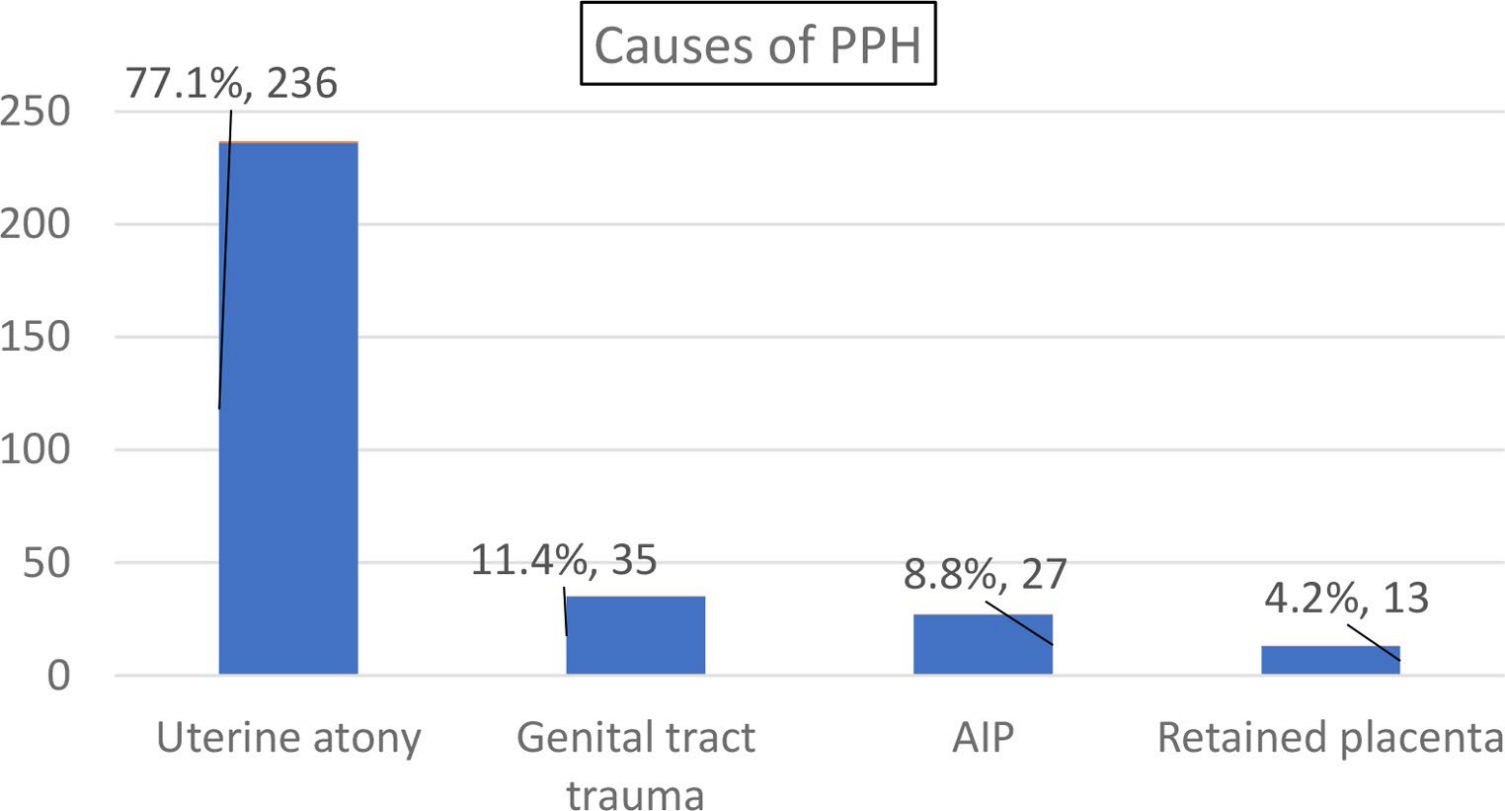
Causes of PPH among women admitted with PPH to hospitals in eastern Ethiopia, 2022 (*n* = 306) *Keys:* PPH: postpartum hemorrhage; AIP: abnormally invasive placenta

The incidence of PPH during the study period was 8 (95% CI: 7–9) per 1000 births (306 in 38,782 births). In almost eight out of ten women (236/306;77%) PPH was primarily caused by uterine atony (Fig. 1).

All women with PPH (*n* = 306), received at least one of the available PPH management options, and 48 had received more than one. Eight in ten(81%) of them received at least one uterotonic drug (oxytocin, ergometrine or misoprostol). Of 236 women with uterine atony, 85% (*n* = 200) received therapeutic oxytocin. Manual removal of the placenta or a placental remnant was done for all the 13 women in whom a retained placenta or placental tissue was identified as a cause (Table 3). Furthermore, uterine atony was the leading cause of PPH in 15 of the 19 maternal deaths from PPH. Regarding interventions for women who died from PPH, 14/19 (74%) received therapeutic uterotonic drugs (oxytocin and ergometrine) (Table 4).

**Table 3:** Interventions for women admitted with PPH at hospitals in eastern Ethiopia, 2022, (n = 306)

Managements	Causes of PPH			
	Uterine atony (n = 236)	Genital tract trauma (n = 35)	AIP (n = 27)	RP/tissue (n = 13)
Oxytocin	200	15	18	8
Ergometrine	26	-	2	2
Misoprostol	5	1	1	1
Manual removal of RP or tissue	6	-	5	13
Intra-abdominal packing	2	1	-	-
B-Lynch suture	7	17	2	1
Hysterectomy	9	2	2	-

*Keys*: RP, retained placenta; NASG, non-pneumatic anti-shock garment; AIP: abnormally invasive placenta

**Table 4:** Interventions for women who died from PPH at hospitals in eastern Ethiopia, 2022, (n = 19)

Managements	Causes of PPH among women who died		
	Uterine atony (n = 15)	Genital tract trauma (n = 2)	AIP (n = 2)
Oxytocin	11	-	1
Ergometrine	2	-	-
Repair of genital tract tear	-	2	-
Hysterectomy	5	-	1

*Keys*: RP, retained placenta; NASG, non-pneumatic anti-shock garment; AIP: abnormally invasive placenta

**Fig. 2:**
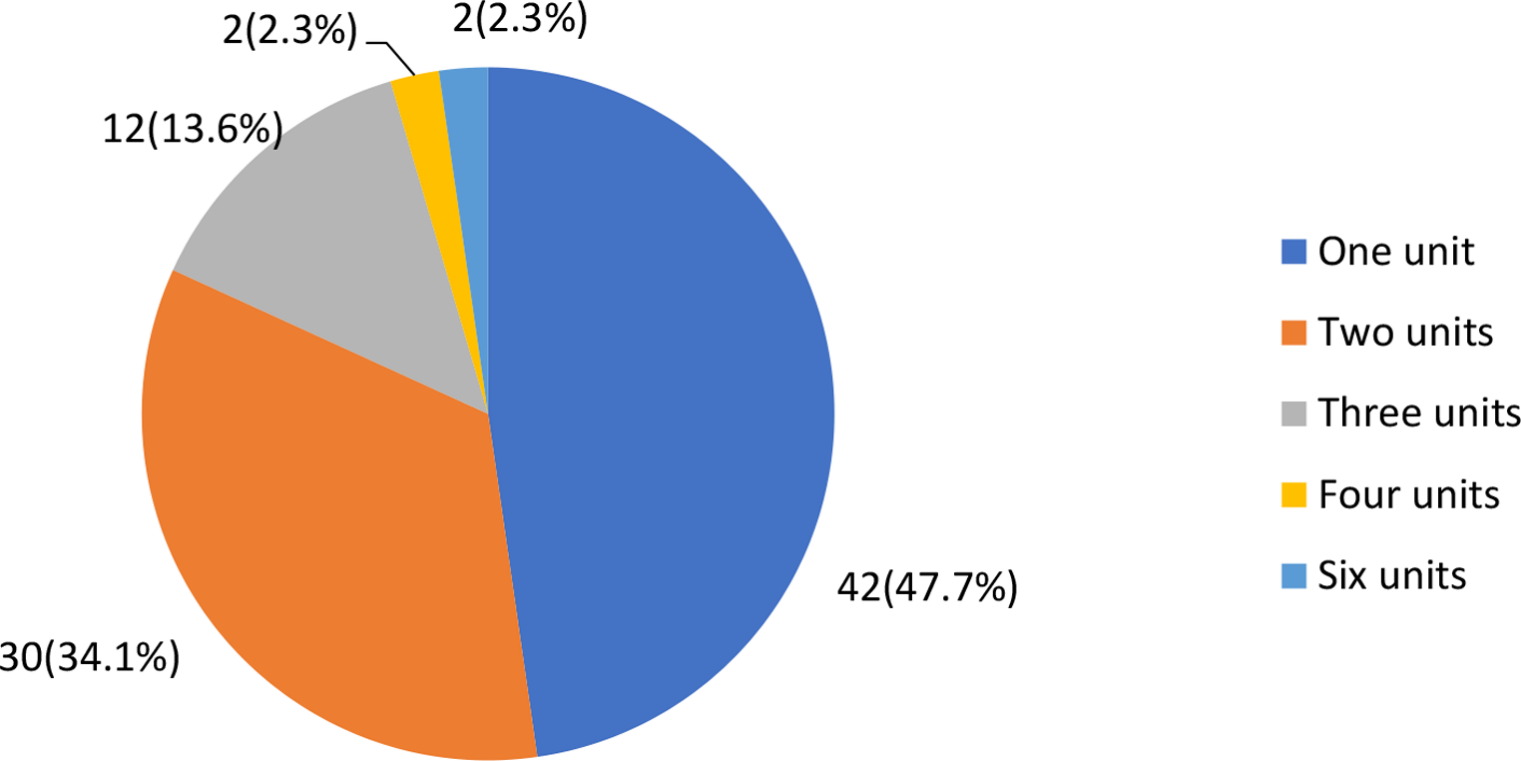
Number of units of blood transfused for women with PPH in eastern Ethiopia, 2022 (*n* = 88)

From 122 (122/306; 40%) women for whom blood was requested for transfusion, only 72% (88/122) received at least one unit of blood. In 82% (72/88) women, only one or two units of blood were transfused. In the remaining 184 (60%) women, no request for blood was made or no documentation was found whether blood for transfusion was requested or not, or given (Fig. 2).

Active management of third stage of labor (uterine massage, uterotonics and controlled cord traction) to prevent PPH was documented in 169 (55%) women. More specifically, of 236 women in whom uterine atony was identified as the primary cause, only 130 (55%) had received active management of the third stage of labor. Of the 306 women admitted to hospitals with PPH, 19 died corresponding with a CFR of 6 per 100. Moreover, PPH was found to be the underlying cause in 27% (19/70) of all hospital-based maternal deaths during the same period.

## Discussion

We estimated the incidence of PPH in public hospitals in eastern Ethiopia as part of the EthOSS project (Abera et al., 2023a). We found the incidence of PPH to be 8 per 1000 births, with a CFR for PPH of 6%. Uterine atony was the underlying cause in the majority of women with PPH. Despite the overall apparently low incidence, PPH was found to be the cause of death in a quarter of all hospital-based maternal deaths during the study period. To the best of our knowledge, this is the first regional multicenter prospective hospital-based obstetric surveillance study of PPH in a low-resource setting in sub-Saharan Africa.

The incidence of PPH in our study was lower than findings from single-center studies in Ethiopia: 3–17% (Achamyelesh & Nebiyu, 2021; Amanuel et al., 2021; Bewket et al., 2022a; Biruk et al., 2019; Habitamu et al., 2019; Sinetibeb et al., 2021) and Zimbabwe (2%) (Ngwenya, 2016). Variations between these studies might be related to possible underreporting of PPH in the current study. From our discussions with obstetric care providers during review meetings and progress reports, it has been repeatedly described that birth attendants and clinicians are too occupied by the focus on lifesaving treatment during these emergencies with little attention to documentation and record-keeping. Moreover, the incidence of PPH we found is also lower compared to reports from high-income countries such as Wales (8.6%) (Bell et al., 2020) and the Netherlands in 2013 (6.4%) (van Stralen et al., 2016). This gap could be due to differences in study participants’ characteristics, definitions of PPH and/or methods used for quantifying or estimating blood loss. We relied on the clinician’s diagnosis of PPH based on visually estimated blood loss, while quantitative blood loss measurements might have been used in those studies from high-income settings. Visual estimation of blood loss is inaccurate in determining amount of blood loss (Prasertcharoensuk et al., 2000; Razvi et al., 1996), and could have contributed to the very low rate of PPH found in our study.

Although we were proactive in putting efforts to reduce the undercount by instituting prospective case identification through daily registration by an assigned midwife in each labor ward, underreporting of PPH in this paper-based surveillance system might happen where details for women admitted or managed for PPH in the hospitals were not properly documented on the admission-discharge, operating theatre, intensive care unit, or birth registers. Similar documentation and record keeping challenges were previously encountered and reported in one of the hospitals included in this surveillance study (Abera et al., 2020).

In line with previous studies (Biruk et al., 2019; Ngwenya, 2016; Sinetibeb et al., 2021), uterine atony was the main underlying cause of PPH in more than three quarter of the women included in this study. We found that the management of PPH, including the use of uterotonics and active management of third state of labor, could be improved. Low use of these management options was particularly evident among women who died. Although prophylactic oxytocin and active management of the third stage should be part of routine immediate postpartum care (Escobar et al., 2022; Federal Ministry of Health of Ethiopia, 2021b, 2022), further research is required to explore whether these are genuinely less frequently practiced than recommended, or whether their use is not well documented.

In this setting with reported shortage of blood for transfusion, both the proportion of blood requested for transfusions and the number of units of blood transfused were low. A low number of transfusions below the required units of blood, might reflects lack of available blood or blood products which could leads to refraining from requesting blood or only request fewer units than actually needed. Ineffective blood transfusion services and lack of voluntary blood donors were common problems reported in other similar settings (Barro et al., 2018; Bates et al., 2008; Getie & Wondmieneh, 2020; Osaro & Charles, 2011; Taye et al., 2017).

Despite many strategic initiatives (Federal Ministry of Health of Ethiopia, 2021b) and interventions such as improved access to Comprehensive Emergency Obstetric Care (CEmOC), and presence of an updated PPH prevention and treatment protocol in Ethiopia (Federal Ministry of Health of Ethiopia, 2021a, b, 2022) the fact that more than a quarter of all hospital-based maternal deaths occurred due to PPH during the study period were due to PPH illustrates that PPH continued to be a major cause of death of women in the study settings. Several interventions have been shown to reduce PPH and its resulting maternal deaths (Bibi et al., 2007; Dupont et al., 2011; Knight & Tuffnell, 2018; Moodley et al., 2014; Seim et al., 2023; Shakespeare & Knight, 2017; Willcox et al., 2020). A care bundle combining interventions was recently shown to have a particularly positive effect (Gallos et al., 2023). This might partly be due to considerable deficiencies in care at baseline: among women in this study 74% had no antenatal care consultations attended, and 45% of women with PPH due to uterine atony didn’t get active management of the third stage of labour. Lack of antenatal care consultations might increase the risk of PPH by letting risk factors go undetected prior to birth (Federal Ministry of Health of Ethiopia, 2021a; Liu et al., 2021). Delays in identifying and referring women at risk of PPH were previously identified as barriers to receiving appropriate and quality care (Bewket et al., 2022a).

A strength of our study is the inclusion of different levels of hospitals, ranging from a rural primary hospital to a tertiary/academic hospital, which helps to obtain a comprehensive perspective on clinical practice across facilities. It should, however, be noted that this study has some limitations. Firstly, blood loss was not quantified and/or estimates were not documented in these settings, and we relied on paper-based hospitals’ charts for the diagnosis of PPH and registers or log books to identify cases of PPH, which might result in under diagnosis or not reporting mild PPH. We also described interventions for women who survived and those who died from PPH based on what is documented in women’s medical records, but were unable to evaluate the timeliness of the interventions due to no or poorly recorded time. Secondly, although we tried our best to include all women hospitalized with PPH through review of all available hospital records, women with PPH might have been missed if they were not included in at least one of these registers, since there is no other mechanism to trace such women. These limitations indicate need for instituting an electronic client chart and record keeping system to improve medical records and registers, to optimize our surveillance system.

While the overall low incidence of PPH might be partly explained by under-reporting, the high CFR indicates that under-reporting is likely limited to less severe cases. With the current stagnation in reducing maternal deaths in general and deaths from PPH in particular, meeting the 2030 Sustainable Development Goals target requires a paradigm shift: implementations of the WHO’s road map to combat PPH (World Health Organization, 2023) through research, appropriate context-specific interventions, monitoring and evaluation, and benchmarks for PPH protocols, timely use of uterotonics, tranexamic acid, and procedural placement reduces the risk of severe haemorrhage sequela and the need for additional procedures (Federspiel et al., 2023). There is a need for audit of care to evaluate its quality, identify delays, and design tailored interventions to improve care (Sørensen et al., 2022).

## Electronic Supplementary Material

Below is the link to the electronic supplementary material.

Supplementary Material 1

## Data Availability

Anonymized data is available from the corresponding author upon a reasonable request.

## Code Availability

Coding is available upon request.
